# Influenza A Virus M2 Protein: Roles from Ingress to Egress

**DOI:** 10.3390/ijms18122649

**Published:** 2017-12-07

**Authors:** Rashid Manzoor, Manabu Igarashi, Ayato Takada

**Affiliations:** 1 Division of Global Epidemiology, Research Center for Zoonosis Control, Hokkaido University, Sapporo 001-0020, Japan; igarashi@czc.hokudai.ac.jp (M.I.); atakada@czc.hokudai.ac.jp (A.T.); 2 Global Station for Zoonosis Control, Global Institution for Collaborative Research and Education, Hokkaido University, Sapporo 001-0020, Japan; 3 School of Veterinary Medicine, The University of Zambia, Lusaka 10101, Zambia

**Keywords:** influenza A virus, M2 protein, pathogenicity

## Abstract

Influenza A virus (IAV) matrix protein 2 (M2) is among the smallest *bona fide*, hence extensively studied, ion channel proteins. The M2 ion channel activity is not only essential for virus replication, but also involved in modulation of cellular homeostasis in a variety of ways. It is also the target for ion channel inhibitors, i.e., anti-influenza drugs. Thus far, several studies have been conducted to elucidate its biophysical characteristics, structure-function relationships of the ion channel, and the M2-host interactome. In this review, we discuss M2 protein synthesis and assembly into an ion channel, its roles in IAV replication, and the pathophysiological impact on the host cell.

## 1. Introduction

Influenza A viruses (IAVs) are RNA viruses belonging to the *Orthomyxoviridae* family. The IAV genome consists of eight segments of negative-sense viral RNA (vRNA), and each segment is complexed with the trimeric viral polymerase proteins (PB2, PB1 and PA) and nucleoprotein (NP) to form viral ribonucleoprotein (vRNP) particles [1]. To date, the approximately 13 kb genome is known to encode 18 different proteins, and only hemagglutinin (HA), NP and neuraminidase (NA) genes so far are known to encode single viral proteins [2,3,4,5,6,7,8,9,10,11,12,13,14,15,16]. The matrix (M) protein gene produces 4 mRNAs: mRNA 1 is an unspliced transcript, and mRNAs 2–4 are spliced transcripts. The mRNA 1 has three 5′ alternative splice sites (5′ ss), first one at position 12 producing mRNA 3, second one at position 52 which produces mRNA 2, and the third one at position 146 which produces mRNA 4. Interestingly, all three-mRNA species undergoing alternative splicing share a common 3′ splice site [7,14,17,18,19]. Unspliced mRNA 1 encodes the M1 protein while spliced mRNA 2 encodes the M2 protein [15], and mRNA 4 encodes the M42 protein [7]. The mRNA 3, produced from the most proximal 5′ ss, is known to encode no protein and is not required for virus growth in tissue culture [20,21,22].

Though primarily an ion channel, the M2 protein is not only involved in various steps of viral replication but also affects the host cellular functions in ion channel activity-dependent and -independent manners. Here, in this review we discuss the fundamental biochemistry and pathobiological roles of the M2 protein in the IAV life cycle from virus entry through synthesis, assembly, and virus release.

## 2. Structure and Function of the M2 Ion Channel

**Figure 1:**
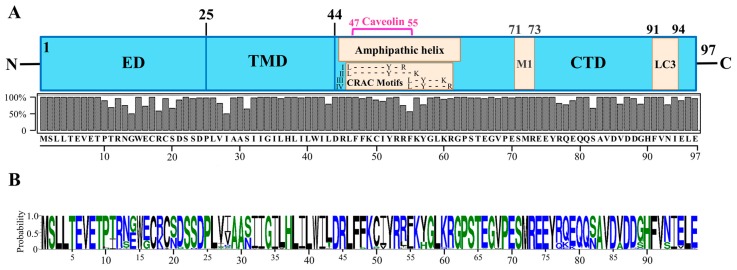
M2 protein structure. (**A**) Domain structure of the IAV M2 protein showing the ectodomain (ED), transmembrane domain (TMD) and C-terminal domain (CTD). The CTD contains important functional and protein-interacting regions. The CTD region from amino acid residues 45–62 forms an amphipathic helix (APH) (adopted from refs. [34,35]). The APH region also contains a caveolin interacting region (residues 47–55) (adopted from ref. [36]), and cholesterol-recognition/interaction amino acid consensus (CRAC) motifs (residues 46–61: four possible CRAC motif sequences are shown) (adopted from ref. [34]). The CTD also contains an M1-interacting region (residues 71–73) (adopted from refs. [27]), and LC3-interacting region (residues 91–94) (adopted from [37]). The M2 amino acid consensus sequence is shown at the bottom. The consensus sequence was determined by aligning 6413 M2 amino acid sequences using UGENE software (version 1.27.0). The M2 amino acid sequences were downloaded from GenBank using the “collapse identical sequences” option. (**B**) M2 protein amino acid sequence logo generated using the WebLogo 3 online tool [38,39].

The M2 protein is a single-pass, type III integral membrane protein (nomenclature of Von Heijne). It is also classified as a class I (subclass IA) viroporin [23,24,25]. Each monomer of the M2 protein consists of 97 amino acids, which are divisible into an N-terminal ectodomain (ED, residues 1–24), middle transmembrane domain (TMD, residues 25–43), and C-terminal domain (CTD, residues 44–97) comprised of an amphipathic helix (APH, residues 45–62), and cytoplasmic tail (CT, residues 63–97) [26,27,28,29,30,31]. Though the M2 protein structure is relatively conserved, its regions from amino acid residues 10 to 28, 54 to 57 and 77 to 93 show relatively higher sequence variations, whereas His37 and Trp41 in all IAVs and first nine amino acid residues in all non-bat IAVs are absolutely conserved (Figure 1) [32,33].

**Figure 2:**
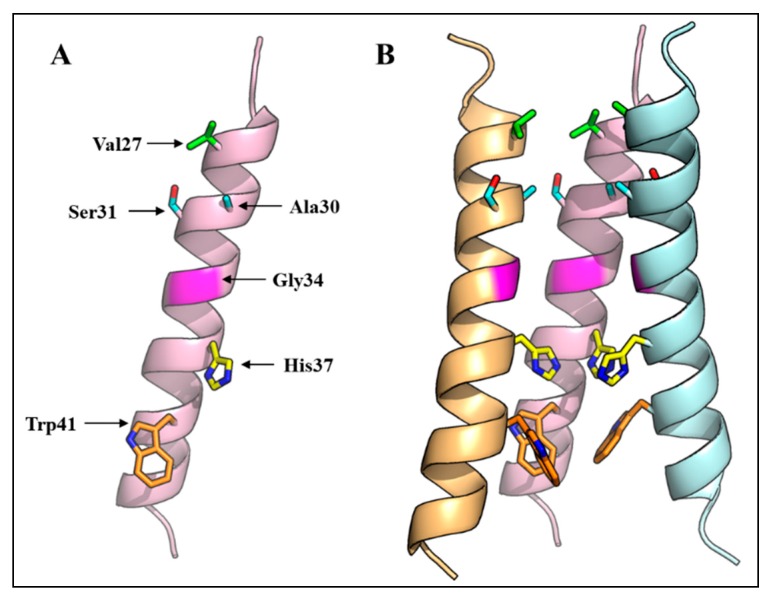
Three-dimensional structure of the IAV M2 ion channel. (**A**) A monomer of the IAV virus M2 protein TMD showing amino acid residues that face the ion channel; (**B**) Three-dimensional structure of the M2 ion channel showing the arrangement of four transmembrane domains, and the orientation of pore-lining residues. One M2 monomer is removed to reveal the side chains of the pore-lining residues. The NMR structure with PDB ID 2RLF was used.

The M2 protein forms a pH-regulated, proton selective ion channel. The channel is activated by low exterior pH reaching its limiting rate at pH 4.5–5.0. The channel is 10^6^–10^7^-fold more permeable to protons than alkali metal cations such as Na^+^ and K^+^ [40,41,42,43,44]. This cation permeability allows the ion channel to function as an antiporter facilitating the efflux of cations (K^+^ and Na^+^) along with proton influx [45]. The selective ion channel activity is dependent upon the ion channel formed by the four alpha helices of TMDs, whose polar and single charged residues are oriented towards the channel and hydrophobic residues are directed towards the lipid bilayer. Each alpha helix shows a helix 3.6 structure from residues 26–43 of TMD [46]. The N-terminal end of the ion channel is constricted by the hydrophobic side chain of Val27; then the pore size of the channel gradually increases until Gly34. At the C-terminal end, the pore is occluded at Trp41. The His37-Trp41 residues form the functional core of the M2 ion channel, in which His37 acts as a proton sensor and conducts protons by protonation/deprotonation of its imidazole side chain while the Trp41 side chain acts as a gate of the channel [31,47,48,49], though a recent study suggests that Asp44 also constitutes an integral part of the channel gate [31]. Various studies employing computational modeling and simulations, cysteine mutagenesis and disulfide cross linking have suggested that the M2 pore is lined by residues 27, 30, 31, 34, 37 and 41 (Figure 2) [50,51,52,53,54,55]. Among these pore-lining residues, non-hydrophobic residues (N/S, G/E and H) are located at positions 31, 34 and 37. Two proton conduction mechanisms have been proposed: the “water wire model” and “proton relay model”. According to the water wire model, a water column in the pore is discontinued at the gate closed by deprotonated His37 residues. However, electrostatic repulsion resulting from the protonation of two or more His37 residues opens up the gate, and makes the water column continuous through which protons are transferred [56,57,58,59]. According to the proton relay model, the His37 imidazole side chain binds a proton from one side (outside) of the gate and releases the already bound proton on the other side (inside) of the gate [53,54,60].

Integral membrane proteins generally prevent the exposure of their hydrophobic TMD regions to the polar environment (hydrophobic mismatch) either by tilting or a kink, usually at Gly residues in their TMDs [61,62,63,64]. In the case of M2, both a TMD tilt (a range of tilt angles of 15°–38°) and a kink at Gly34 have been reported. Factors such as membrane thickness, composition, pH, and drugs such as amantadine and rimantadine, have been shown to modulate both the tilt and kink angles [31,46,54,65,66,67,68,69,70,71]. For example, the tilt angle calculated in 1,2-dimyristoyl-sn-glycero-3-phosphocholine (DMPC) micelles (37° ± 3°), was 4° more than that calculated in 1,2-dioleoyl-sn-glycero-3-phosphocholine (DOPC) micelles (33° ± 3°) which are thinner (23Å) than DOPC micelles (27 Å) [65,67,68,69]. The drug amantadine was shown to affect the kink angle at Gly34. In the absence of the drug at high pH, a broad range of kink angles (no kink to 50°) were observed. However, the range of kink angles at Gly34 became narrow (~10°) in the presence of amantadine, making the M2-TMD ion channel population (with respect to kink angle) conformationally more homogenous. The amantadine-binding site is formed by pore lining residues Val27, Ala30/Ser31, and Gly34. The interaction of amantadine with pore limning residues limits the conformational flexibility of M2-TMD which is required for functioning of many channel proteins, and this limited conformational flexibility results in an increase in homogeneity in both tilt and kink angles [46,70,72,73].

Though the structures of TMD and APH helices are well studied, the structures of the ED and CT are largely unknown. Recent studies show that ED is an intrinsically disordered random coil with significant mobility that decreases near the TMD. These studies suggest that the ED conformation depends on the membrane composition. In DMPC bilayers, some residues show a β-strand conformation that shifts to an α-helical conformation in the presence of cholesterol (mimicking the viral membrane) [74,75]. It is worthwhile to mention that crystallography of M2 ED—Fab complex also suggested that M2-ED exists in different conformations supporting the notion that M2-ED is intrinsically disordered [76,77]. The post-APH CT up to residue 71 is largely unstructured with random coil conformation, highly mobile and, unlike the ED, insensitive to the membrane composition [75,78]. Together, these studies suggest that the presence of either the ED or CT favors TMD conformation towards a more stable, resembling drug bound state [74,75,78]. Moreover, the post-APH CT also changes the TMD conformation to one favoring proton binding, though its deletion does not affect the ion channel activity [74].

## 3. Release of vRNP

The entry of IAVs into cells starts from binding of HA to terminal sialic acid residues of sugar chains on host cell surface glycoproteins and glycolipids [79]. This binding initiates a cascade of events resulting in clathrin-dependent or -independent endocytosis of virus particles [80,81,82]. These events do not ensure successful infection, yet the virus must release its vRNPs (replication machinery) into the host cell. While passing through endosomal compartments, the M2 protein plays a crucial role. Recent studies suggest that vRNP release is a stepwise process resulting from stepwise priming of the virus core, which is stabilized by M1-M1, M1-virus envelope, and M1-vRNP interactions [83,84].

The early endosome environment, pH 6.0–6.5, and high Na^+^ and low K^+^ concentrations, activate the M2 ion channel, resulting in an influx of protons and the efflux of K^+^ from the virus core. Consequently, the virus core is weakly acidified with a possible weakening of M1-vRNP or M1-M1 interaction [85], as indicated by a decrease in stiffness of virus particles by 26% [86]. As the endosomes mature from early endosomes to late endosomes, the virus is exposed to a contrasting environment (pH 5.4–6.0) of low Na^+^ and high K^+^ concentrations. Despite its poor K^+^ conductivity, it is only the late endosome environment that promotes K^+^ influx through the M2 ion channel. At this stage both high K^+^ and low pH act as second priming steps, resulting in dissociation of M1 from the virus envelope and vRNPs [85], indicated by a further 36% reduction in the stiffness of virus particles compared to that of at pH 7.4 [86]. By this time, the HA conformational change takes place, allowing fusion between the viral envelope and endosomal membrane, leading to release of vRNPs [85,86,87]. These findings are further supported by previous studies demonstrating that the C-terminal domain of the M1 protein binds vRNPs at neutral pH, and that M1-vRNP complexes dissociate at acidic pH [88,89].

As stated earlier, there are two proposed proton conduction models, the “water wire model” and “proton relay model”. However, the question of how well the antiporter activity fits in these models needs to be addressed. Furthermore, it is also not known that how does the K^+^ influx occur in an already protonated virus core in the late endosomes?

## 4. M2 Synthesis, Posttranslational Modification, and Transport to Cell Surface

The released vRNPs are transported into the nucleus where viral genome transcription and replication take place. The M2 protein is synthesized in the late phase of viral replication [90,91]. It has been shown that mRNA 2 5′ ss is in a weaker form than that of mRNA 3. As a result, mRNA 3 5′ ss is preferably spliced during early phase of infection, causing a delay in the splicing of mRNA 2 5′ ss till late phase of infection. This mechanism ensures that M2 protein is expressed only when it is required [20,21]. The synthesized M2 protein undergoes three different post-translational modifications: (i) intermolecular disulfide-bond formation at two highly conserved Cys residues at positions 17 and 19 [92,93]; (ii) palmitoylation at Cys50 [92,94,95]; and (iii) phosphorylation at Ser residues (~85% at Ser64 and to a minor extent at Ser82, 89 and 93) [92,96]. Interestingly, none of these posttranslational modifications significantly affect viral replication or M2 ion channel activity [92,97]. The newly synthesized M2 protein is inserted in the endoplasmic reticulum (ER) membrane, and this insertion strictly depends upon the signal recognition particle [98]. The signal recognition particle is a universally conserved ribonucleoprotein that targets specific proteins to the ER membrane [99]. The M2 membrane targeting signal sequences are present in the TMD, since removal of at least two amino acid residues from the TMD markedly reduces the membrane integration of the M2 protein [98].

Newly synthesized viral proteins are transported to their final destinations via a well-organized, interconnected network of cellular organelles and transport proteins. In the case of IAV, very detailed studies have been conducted to determine the roles of host factors in the transport of vRNPs, HA, and NA from their synthesis sites to the virus budding sites [100,101,102]. Recently, it has been reported that the cell surface expression of the M2 protein is modulated by some host factors such as transport protein particle complex 6A (TRAPPC6AΔ), ubiquitin protein ligase E3 component N-recognin 4 (UBR4) and Rab11. TRAPPC6AΔ is isoform 2 of the TRAPPC6 gene and is a component of multimeric TRAPP complex primarily involved in ER-to-Golgi transport [103,104]. The M2 protein interacts with TRAPPC6AΔ through a highly conserved L96 residue in the CT region [105]. UBR4 shows both nuclear and cytosolic localization, and is involved in diverse functions such as membrane modeling, cytoskeleton dynamics, ubiquitination, etc. [106]. The M2 protein interacts with UBR-4 through the M2-TMD and CT regions [107]. Rab11 is localized in post-Golgi vesicles and recycling endosomes, and involved in the late recycling process [108]. It seems that TRAPPC6Δ reduces, whereas Rab11 and UBR4 both increase, the M2 surface expression since knockdown of TRAPPC6Δ increases and knockdown of Rab11 and UBR4 reduces M2 surface expression. These proteins appear to transport the M2 protein with or without IAV surface glycoproteins, since knockdown of TRAPPC6Δ, Rab11 and UBR4 exhibits no effect, an increase, and a decrease in the HA surface expression, respectively [105,107,109]. These findings suggest that, at some point en route to the plasma membrane, M2 transport is separated from that of HA and NA proteins. However, convincing evidence requires further studies.

## 5. Assembly into Tetramer

Monomers of M2 are synthesized cotranslationally, and their assembly into functional ion channels takes place in a dynamic membrane environment. Both covalent and noncovalent interactions in the M2 molecule participate in holding the oligomeric proteins in functional conformations. Two disulfide bonds at Cys17 and 19 hold the M2 monomers into dimers [93,96]. Interestingly, none of the residues seem to be essential for viral replication, and noncovalent interactions are sufficient to form an ion channel [110]. This finding is further supported by the fact that a 25-residue peptide (residues at positions 22–46), not having both Cys17 and 19 residues, can assemble into a functional proton transporter [28,111,112,113]. Therefore, it is plausible that the high surface expression of the M2 protein also facilitates this noncovalent assembly into functional tetramers.

In fact, M2 tetramer assembly is modulated by many factors such as membrane composition, pH, and even the M2-ED and CT structures. A low peptide/lipid molar (P/L) ratio favors the predominance of dimers, whereas a high P/L molar ratio favors the predominance of tetramers [114,115]. The assembly of the M2 tetramer seems to be a sequential process; first monomers assemble into dimers, then two dimers associate into a tetramer termed a dimer of dimers [115]. This tetrameric structure is further stabilized by deep insertion, and orientation of the polar and hydrophobic residues of the M2-APH in a way that the hydrophobic residues interact with lipid tails and hydrophilic residues interact with polar head groups of membrane lipids [116,117,118].

## 6. M2 Intracellular Trafficking and Its Impact on Host Cells

### 6.1. Ion Channel Activity-Dependent Pathobiological Effects

Cellular secretory pathways consist of rough endoplasmic reticulum (rER), rER exit sites, rER-to-Golgi intermediate compartments, the Golgi complex, and post Golgi organelle/carriers carrying cargo to predetermined destinations. These constituents of secretory pathways are well-organized structures capable of providing suitable environments for protein folding, post-translational modification, and sorting to their final destinations [119]. One requisite for carrying out these functions is to maintain the right pH level in each compartment.

While moving along a trans-Golgi network (TGN) whose pH is sufficiently low, the M2 ion channel becomes active and raises the lumenal pH of the TGN [120,121]. This alkalinization in the secretory pathway affects not only cell activity but also homeostasis in various ways. The M2 protein elicits the amantadine-sensitive cytotoxicity observed in insect cells [122], *Escherichia coli* [123], *Saccharomyces cervisiae* [124], and Human embryonic kidney 293 cells [125]. The ion channel activity-dependent pH perturbation also significantly delays both intra-Golgi transport and cell surface delivery of cargo proteins such as the 70% reduction seen for cell surface delivery of HA in M2-expressing cells [126]. Other studies suggest that the M2-mediated pH perturbation is specific to early endosomes and the TGN of secretory pathways, and typically affects apical membrane trafficking of proteins [121,127].

**Figure 3:**
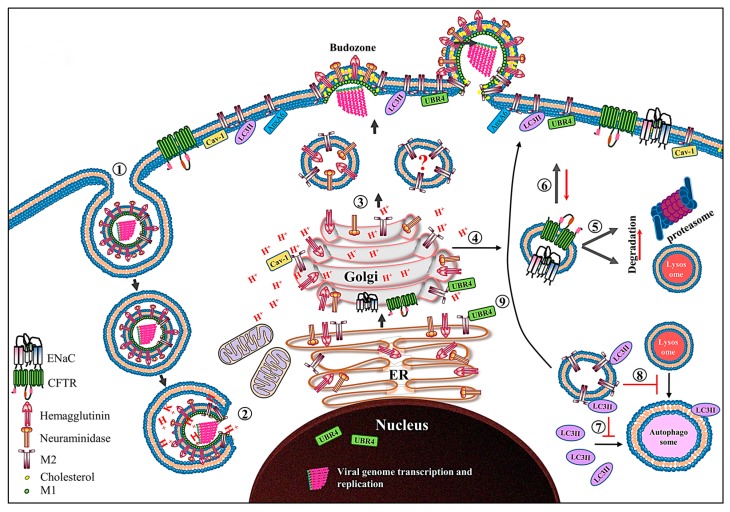
Schematic diagram showing the role of the M2 protein in the IAV life cycle. After receptor-mediated endocytosis ➀, the low pH in the endosome primes and dismantles the virus core, resulting in release of the vRNPs in cytoplasm ➁ and their transport into the nucleus where viral genome transcription and replication take place. The newly synthesized viral proteins (only HA, NA and M2 proteins are shown) move from the endoplasmic reticulum (ER) to the Golgi complex and then to the cell membrane. The M2 only transport vesicle is marked with question mark since there is no direct evidence which supports that M2 is transported independent of HA and NA proteins ➂. While passing through the trans-Golgi network (TGN), low TGN pH activates the M2 ion channel. The activated M2 ion channel equilibrates the TGN pH with that of cytoplasm. The M2 ion channel activity ➃ increases the degradation of CFTR and ENaC transporter proteins by either lysosomal or proteasomal degradation pathways ➄, and also reduces their surface expression ➅. The M2 protein prevents the association of LC3II protein with autophagosomes ➆ and thereby prevents the fusion of lysosomes with autophagosomes ➇. Moreover, the M2 protein relocalizes the LC3II protein to the cell membrane ➈. Caveolin-1 (cav-1), ubiquitin protein ligase E3 component N-recognin 4 (UBR4) and annexin A6 (AnxA6), which interact with M2 protein are also shown.

The pH change in secretory pathways has been shown to affect the activities of two voltage-gated proton channels, human cystic fibrosis transmembrane conductance regulator (CFTR) and amiloride-sensitive epithelial sodium channels (ENaC). CFTR is a cyclic AMP-activated chloride (Cl^−^) channel in lung epithelium that helps regulate the thickness and composition of lung epithelium-lining fluids. The pH increase in secretory pathways inhibits the CFTR activity by redirecting it to the ubiquitin-mediated proteasomal or lysosomal degradation system [128,129]. The M2 protein gradually increases intracellular levels of reactive oxygen species, possibly by altering the membrane potential across organelles such as mitochondria, that stimulate protein kinase C, increasing the endocytosis and proteasomal degradation of ENaC, culminating in reduction of its apical membrane levels and function (Figure 3) [130]. The volume and composition of epithelial-lining fluids are maintained via a delicate functional balance between the secretion and absorption of fluids and electrolytes by Cl^−^ and Na^+^ channels, respectively; and both types of channels are present in apical membranes of lung epithelium [131,132]. Therefore, the M2-medated perturbation in the function of CFTR and ENaC is thought to be one of the factors leading to rhinorrhea and lung edema, causing exacerbation of respiratory pathology [128,129,130]. Another consequence of the M2-mediated perturbation in Golgi pH is activation of the NLRP3 inflammasome in dendritic cells and macrophages. The NLRP3 inflammasome is one of the key innate immune system sensors that regulate the activation of caspase-1, which in turn cleaves proinflammatory cytokines such as pro IL-1β and pro IL-18 into their bioactive forms [133]. The NLRP3 inflammasome-mediated cytokine release requires two signals. In case of IAV, signal 1 is provided by TLR7 activation, while signal 2 is provided by the M2-midiated transport of protons from acidified Golgi. Interestingly, NLRP3 inflammasome activation is not solely dependent upon H^+^ transport since the His37Gly substitution in TMD, which results in the loss of M2 proton selectivity and enables the transport of other cations (Na^+^ or K^+^), causes almost 2-fold more activation of the NLRP3 inflammasome than wild-type M2 [134]. These findings suggest that any imbalance in the cation concentrations can lead to the NLRP3 inflammasome activation.

### 6.2. Ion Channel Activity-Independent Pathobiological Effects

In addition to its ion channel activity-dependent effects, the M2 protein potentially affects host cell conditions by directly interacting with many host proteins. While en route to the cell membrane, the M2-ED and CTD are exposed to vesicular/organellar and cytoplasmic environments, where they may interact with various cellular proteins [135].

It is well known that IAV infection not only triggers the formation of autophagosomes but also inhibits the fusion of autophagosomes with lysosomes. Upon activation of autophagy, the microtubule-associated protein 1 light chain 3 protein (LC3) is cleaved by the cysteine protease Atg4 to form LC3-I, which is lipidated to form LC3-II. LC3-II is required for fusion of autophagosomes with lysosomes, creating autophagolysosomes and resulting in cargo degradation [136,137,138]. The M2 protein prevents the fusion of autophagosomes with lysosomes, resulting in accumulation of autophagosomes in the cells [139,140]. The C-terminal end of the M2-CT region (residues 91–94) contains the LC3-interacting region (LIR), which binds to the LC3/Atg8 protein and relocalizes it to the plasma membrane, thereby preventing the fusion of autophagosomes with lysosomes (Figure 3) [37,141].

The M2-APH region possesses a caveolin-1 (cav-1)-interacting motif/region. Though cav-1 is a cholesterol-rich raft-residing protein, the interaction between cav-1 and M2 is cholesterol independent. Cav-1 is a membrane scaffolding protein recruiting cargo proteins such as signal transduction proteins and viral proteins to lipid rafts [142]. The reported consensus cav-1-interacting motif in M2 is ΦxxxxΦxxΦ, where Φ denotes any of the aromatic amino acid residues at positions 47, 52 and 55. The M2/cav-1 interaction modulates IAV replication but the exact molecular mechanisms are not yet known [36,143].

Double-stranded RNA (dsRNA)-activated protein kinase (PKR) is an interferon-induced protein kinase, that is activated by autophosphorylation after binding with dsRNA or protein activators of PKR [144]. IAVs exploit PKR in two ways for propagation. During the early phase of infection, IAVs prevent PKR activation by synthesizing viral nonstructural protein 1 (NS1), which masks the viral dsRNA. Moreover, viral NP displaces Hsp40 from the Hsp40-P58^IPK^ complex, thereby releasing P58^IPK^, a PKR inhibitor, and promoting viral replication [145,146,147]. During the late phase of infection, the M2 protein associates with the Hsp40-P58^IPK^ complex and prevents their dissociation, resulting in the activation of PKR, with resultant cell death and virus release [148]. Therefore, this NS1/M2-mediated sophisticated PKR control favors both viral replication and virus release.

The Cysteine Aspartate-Specific protease (caspase) cleavage motifs have been identified in proteins of many viruses [149]. In the case of M2 of IAVs, two caspase cleavage motifs have been identified, first in the ED (DSSD_23↓_P) and the second one (VDVDD_87↓_G) in the CT. It was shown that alterations in either N- or C-terminal caspase cleavage motifs of a highly pathogenic AIV affected the virus replication potential and pathogenicity for chickens in different manners. The mutant virus with mutation in the N-terminal caspase motif displayed reduced replication in cultured cells and apathogenicity for chickens, while the mutant virus with mutation in the C-terminal caspase motif showed unaffected replication in cultured cells and significant reduction in pathogenicity for chickens [150].

## 7. Virus Budding and Scission

In contrast to many other enveloped viruses, the budding and scission processes of IAVs are ESCRT independent and mediated by the M2-APH region [109]. The amphipathic helices are generally known membrane curvature generators and sensors [151,152]. In the case of IAV M2 protein, a peptide (17 amino acid residues) corresponding to the APH region has been shown to induce budding from giant unilamellar vesicles containing 0.5 molar % cholesterol [109]. The M2-APH region (residues 45–62) has two unique features, a palmitoylated Cys50 residue [92,95] and a cholesterol recognition/interaction amino acid consensus (CRAC) motif (L/V-X1-5-Y-X1-5-R/K) [153]. The CRAC motif is responsible for cholesterol binding and membrane association, whereas palmitoylation at Cys50 is responsible for M2 association with lipid raft regions, both of which are required for efficient membrane binding [34]. It seems that other regions of the M2 protein also act cooperatively to facilitate virus budding and scission since the M2-CT region (containing APH) generates negative Gaussian curvature (NGC) to a lesser extent than the full length M2 protein [34,154]. Studies using solid state NMR confirmed that the M2-APH region altered the membrane curvature in a cholesterol-dependent manner as seen with unilamellar vesicles [109,155]. Some in vivo studies also demonstrated that viruses having mutations in the APH region exhibited classical “beads on string” phenotypes, suggesting impaired scission and release of the virions [34,35,109]. Taken together, both in vivo and in vitro studies suggest that the M2-APH region plays pivotal roles in virus budding and scission.

Annexin A6 (AnxA6) is a member of Ca^++^ dependent membrane binding proteins. It is known to interact with membrane phospholipids, cholesterol-rich lipid rafts and F-actin, enabling it to act as a membrane scaffolding protein [156]. Although IAV budding is ESCRT independent, it was recently shown that AnxA6 could modulate the budding of IAVs. Knockdown of AnxA6 increased virus release, whereas its overexpression reduced virus release from infected cells. Based on the results, it was proposed that AnxA6 interacted with the M2-CT region and interfered with the M2-mediated scission of budding virions by directly inhibiting the M2 function, modifying the membrane environment or modulating some host protein functions involved in viral budding or all [157].

The HA and NA proteins associate with lipid raft microdomains, and these rafts coalesce into larger viral budozones, while the M2 protein is arranged at the edges of these budozones [158,159]. The vRNPs are recruited at these sites, and the M1 protein acts as a bridge between M2 and HA proteins. As a bud grows, the M2 protein becomes localized at the neck of the growing bud and constricts the neck of the bud by generating NGC, resulting in the pinch off and release of the virion (see reviews [159,160] for details).

## 8. Future Perspectives

In the case of IAVs, a great deal of attention has been paid to the molecular mechanisms in IAV infection, and host interactomes of viral proteins such as viral polymerases, NP, and NS proteins. However, information on the M2-host interactome and its role in infection has started emerging very recently, thereby requiring further focus on this area. Thus far, conducted studies suggest that IAV M2 protein contributes to the viral pathogenicity in M2 ion channel activity-dependent or -independent manners either by interfering with cellular homeostatic processes or by interacting with host proteome and modulating its functions. It can be speculated that detailed understanding of the M2 protein structure-function relationship, role in virus infection, and roles of host proteins in assisting the M2-mediated functions such as vRNP release and budding can assist in the development of novel antivirals targeting the specific steps of the IAV replication cycle, thereby reducing the potential risk of emergence of drug-resistant IAVs.
